# Effects of NPK and biochar fertilized soil on the proximate composition and mineral evaluation of maize flour

**DOI:** 10.1002/fsn3.808

**Published:** 2018-10-11

**Authors:** Adewale Muyideen Ogunyemi, Bolanle O. Otegbayo, John A. Fagbenro

**Affiliations:** ^1^ Department of Food Science and Technology Bowen University Iwo Nigeria; ^2^ Department of Crop Science and Environmental Studies Bowen University Iwo Nigeria

**Keywords:** biochar (sawdust and gliricidia biochar), maize, mineral evaluation, NPK fertilizer, proximate composition

## Abstract

Series of farming practice methods have been employed to increase maize production but there is no adequate information on the effect of these methods on the nutritional and mineral content of organically grown maize. This study investigated the effects of inorganic and biochar fertilized soils on the proximate composition and mineral content of maize. Maize seeds were planted on organically fertilized soil (sawdust and gliricidia biochar), chemically fertilized soil Nitrogen Phosphorus and Potassium (NPK fertilizer), and soil without any amendment as control. The proximate compositions (protein, ash, crude fat, carbohydrate, and moisture) and mineral contents (Na, Mg, K, Ca, Fe, and Zn) of the maize flour samples were determined using standard methods. The results showed that protein content ranged from 4.58% to 7.24% (protein), ash 0.82% to 1.09%, crude fat 3.84% to 4.61%, moisture 9.76% to 10.60%, and carbohydrate 76.85% to 80.31%. There was no significant (*p* ≤ 0.05) difference among the proximate compositions except for protein and carbohydrate. Maize planted on NPK fertilized soil had the highest crude protein content of 7.24%. Other results obtained included sodium (55.65 mg/100 g), magnesium (35.87 mg/100 g), and iron (6.78 mg/100 g). Maize from soil without amendments was significantly higher than maize from NPK fertilized and biochar fertilized soils. Also, maize from control plot had the highest calcium content value of 48.95 mg/100 g. We concluded that maize planted with NPK fertilizer had higher nutrient than those planted with biochar application. Also, the mineral content of maize planted in control plot was higher than those on the amended soil.

## 1 INTRODUCTION

Maize serves as the main source of dietary energy in Nigeria apart from cassava flakes, rice, wheat, and sorghum. As a multipurpose crop, it can be used in fuel making (ethanol), as feeds for animals (poultry and livestock) and as foods (e.g., agidi, eko, ogi, tuwo, mosae.t.c.)

Maize had been reported to have great nutritional value and can be used as raw material for producing many industrial products (Afzal, Nazir, Bashir, & Khan, 2009). Johnson (2000) reported that maize (*Zea mays*) is the second most widely produced cereal crop worldwide which is produced in the entire world except Antarctica. Maize remains an important part of human diet in many developing countries.

Biochar is a carbon‐rich coproduct resulting from pyrolyzing biomass under high‐temperature, low‐oxygen conditions for biofuel production (Laird, 2008; Lehmann, 2007) and although it is similar to other charcoals, biochar is defined by its intentional application to the soil for environmental applications (Lehmann, 2009). It contains highly condensed aromatic structures that resist decomposition in soil and thus can effectively sequester a portion of the applied carbon for decades to centuries (Lehmann, Gaunt, & Rondon, 2006).

Biochar has been reported to increase the emergence of maize in the field based on its porous nature; it helps to retain soil moisture for a longer period while increasing the relative and absolute growth rates of maize due to its increase mineral availability by increasing the cation exchange capacity (Peng, Ye, Wang, Zhou, & Sun, 2011). Positive effect of biochar as organic fertilizer on crop yield was reported to be mainly attributed to its own nutrients and indirect fertility, thus making it to be referred to as soil fertilizer and soil conditioner, respectively (Glaser, Lehmann, & Zech, 2002). Biochar has been considered as a key input for rising and sustaining production and simultaneously reducing pollution and dependence on fertilizers (Barrow, 2012).

Recently, health conscious consumers are interested in optimizing the nutritional composition of food with minimal chemical residues on foods produced through environmentally friendly agricultural practices (Amujoyegbe, Opabode, & Olayinka, 2007). Various methods of organic soil fertility have been reported to increase the agronomical yield of crops; however, there is a paucity of information on the food quality of the produced through soil fertility by application of organic fertilizer (such as biochar) used in crop production. The study was focused on determining the effects of NPK and biochar fertilized soil on food quality of maize.

## 2 MATERIALS AND METHODS

### 2.1 Materials

Four different maize samples were obtained from the Department of Crop Production, Soil and Environmental Management Bowen University Iwo Osun State. Maize was planted at the Research farm of Bowen University. Saw dust feedstock, which was a mixture of wood waste sawn from indigenous hardwoods of *Triplochitonsceleroxylon*,* Milletiaexcelcia*, Terminalia species, and *Caciasiame*, was collected from Oluwaseyi saw mill within Iwo municipality. *Gliricidia sepium* feedstock was from *Gliricidia sepium*, a nitrogen fixing tree species that grows widely on fallow land in Nigeria.

### 2.2 Methods

#### 2.2.1 Maize planting

The experimental site was located in an abandoned agricultural farm overgrown with *Imperata cylindrical* with a few scattered pawpaw and plantain stands and trees within the University Teaching and Research Farm. The soil is an Oxisol (Aubert & Tavenier, 1972; FAO/UNESCO, 1997). The site was weeded manually and stumped before plowing. The soil within the experimental plot was relatively uniform and was randomly sampled at 10‐m interval at the predetermined depth of 0–30 cm. The experiment was laid out in randomized complete block (RCB) design with three replicates. There were three blocks separated from one another by a 3‐m ride. Each block was made up of eight plots, each plot measuring 4 × 4 m, and was separated from one another (within the block) by a 2‐m space. Four seeds of improved maize variety, OBA SUPER‐2 Hybrid, were sown at a spacing of 75 cm between rows and 50 cm within the row. The emergent seedlings were thinned to two per stand 2 weeks after germination. The maize plants were rain‐fed throughout the experimental period. At maturity, which was approximately 12 weeks, the plants were harvested. All the 12 plants contained within a subplot measuring 1 by 0.75 m located in the middle of each plot were harvested.

#### 2.2.2 Soil analysis

The site was weeded manually and stumped before plowing. The soil within the experimental plot was relatively uniform and was randomly sampled at 10‐m interval at the predetermined depth of 0–30 cm. A composite soil sample made up of 10 auger points was then produced. The sample was air‐dried, sieved though a 2‐mm sieve, and analyzed for selected key physico‐chemical properties (Table 1). The soil reaction (pH) was determined potentiometrically in soil: water ratio of 1:2 using a Kent model 720 glass electrode (pH) meter. Organic carbon was determined using the Walkley and Black (1934) chromic acid digestion procedure. Total N was determined using the method of Keeney and Bremner (1966), while soil available P (Mehlich), K, Ca, Mg, and Na were determined following IITA (1982) routine procedures. The effective cation exchange capacity (ECEC) was determined by adding the values of all the cations and the exchangeable aluminum together. The water holding capacity of the soil was determined to be 44.5%.

**Table 1:** Some properties of soil (0–30 cm) collected from the experimental plot

Properties	Value
Particle sizes	
Sand (g/kg)	872
Silt (g/kg)	40
Clay (g/kg)	88
pH (H_2_0)	6.2
Total N (g/kg)	2.3
Organic C (g/kg)	19.9
Available P (mg/kg)	16.5
Exchangeable cations (cmol/kg)	
K	0.24
Na	0.60
Mg	1.35
Ca	13.50
Exchangeable micronutrients (mg/kg)	
Cu (mg/kg)	2.36
Zn (mg/kg)	5.69
Fe (mg/kg)	78.30
Mn (mg/kg)	79.60

#### 2.2.3 Biochar production

The feedstocks were converted separately to biochars by heating in an engineered gas‐ignition pyrolyser. The average temperature within the pyrolyser was 400°C. Key chemical properties of the two biochars were determined using routine procedures (IITA (International Institute of Tropical Agriculture), 1982) while their humic substances content was exhaustively extracted with 0.1 M NaOH solvent and fractionated according to Fagbenro (1988). These properties are presented in Table 2.

**Table 2:** Selected properties of saw dust biochar (SB) and gliricidia biochar (GB) used for the field experiment

Property	SB	GB
pH (H_2_O)	8.1	8.5
pH (CaCl_2_)	7.8	8.3
N0_3_^−^N (g/kg)	1.7	1.0
NH_4_^−^N (g/kg)	0.8	0.4
Total org. C (g/kg)	908.7	982.8
Total N (g/kg)	11.3	10.3
C:N ratio	80.4	86.7
Total P (g/kg)	3.8	2.8
K (g/kg)	5.4	4.0
Mg (g/kg)	1.9	1.4
Ca (g/kg)	1.5	1.7
Na (g/kg)	1.8	1.4
S (g/kg)	0.9	0.7
Ash (g/kg)	38.0	37.2
HA (g/kg)	80.9	92.2
FA (g/kg)	49.0	41.2
HA:FA ratio	1.65	2.24
Total micronutrients (mg/kg)		
Mn	17.6	9.4
Cu	2.4	9.8
Zn	479.8	14.7
Fe	23.1	9.4
Exch. acidity (c mol/kg)	0.65	0.25
CEC (cmol/kg)	106.38	45.38

*Note*

FA: fulvic acid; HA: humic acid.

#### 2.2.4 Method of fertilizer application

The two biochars were applied at the rate of 2.5 t/ha, while NPK 15:15:15 inorganic fertilizer was applied at the rate of 90 kg N ha^−1^. There were four treatments consisting of (i) control (no amendment), (ii) NPK 15:15:15 inorganic fertilizer (90 kg N ha^−1^), (iii) saw dust biochar alone (2.5 t/ha), and (iv) gliricidia biochar (GB) alone (2.5 t/ha). The organic and inorganic amendments were applied to the soil by broadcasting them evenly within each plot and then mixed manually with soil using a hoe.

#### 2.2.5 Maize flour production

**Figure 1:**
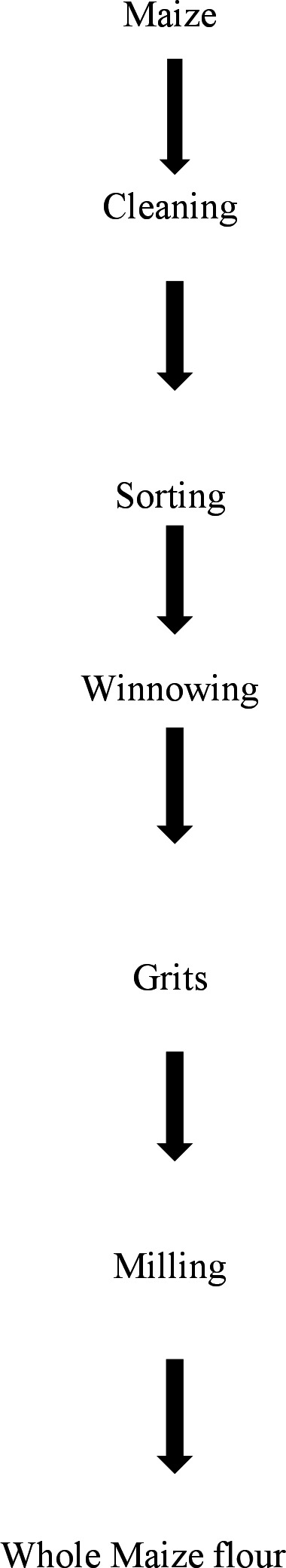
Flowchart of maize flour preparation from maize grains

Modified method of Houssou and Ayernor (2002) was used to prepare maize flour. Yellow maize kernels were sorted to remove stones, dirt, and other foreign materials. Maize kernels were milled by means of Waring Blender HGBTWO, USA, into flour as shown in Figure 1. Maize flour samples were triplicated and labeled A–D where Sample A is maize planted on normal soil (control), Sample B is maize planted on NPK fertilized soil, Sample C is maize planted on sawdust biochar (SB), and Sample D is maize planted on GB.

#### 2.2.6 Proximate composition and minerals

##### Proximate analysis

Maize flour samples were labeled A–D where Sample A is maize planted on normal soil (control), Sample B is maize planted on NPK fertilized soil, Sample C is maize planted on SB, and Sample D is maize planted on GB. Samples were analyzed for proximate composition: protein, moisture, fat, ash, and mineral composition according to the method of AOAC (2005). Carbohydrate content was obtained by difference. All analyses were done in triplicates.

##### Mineral analysis

The samples were ashed at 550°C. The ash was boiled with 10 ml of 20% hydrochloric acid in a beaker and then filtered into a 100 ml standard flask. This was made up to the mark with deionized water. Sodium (Na) and potassium were determined using the standard flame emission photometer. NaCl and KCl were used as standards (AOAC, 2005). Calcium (Ca), potassium (K), magnesium (Mg), and iron (Fe) were determined using Atomic Absorption Spectrophotometer (AASmodel SP9). All values were expressed in mg/100 g.

## 3 RESULTS AND DISCUSSION

The result of the proximate compositions of maize flour samples is in Table 3; the percentage mean crude protein content was in the range of 4.58%–7.24%. There was significant difference among the samples. Maize planted with NPK fertilizer was significantly higher in protein than in the other maize samples. This is in agreement with the report of Matt, Rembialkowska, Luik, Peetsmann, and Pehme (2011), which reported higher protein content in fertilized maize as a result of higher nitrogen fertilization. There was no significant difference in the protein content of maize planted with both sawdust and GB; this may be as a result of the inability of the biochar as organic fertilizer to provide adequate nitrogen required by the maize crop in early application.

**Table 3:** Proximate composition of maize flour

Samples	Protein	Ash	Crude fat	Carbohydrate	Moisture content
A	5.20 ± 1.00^c^	0.97 ± 0.23^a^	3.84^a^ ± 1.00^a^	80.20^b^ ± 0.08^b^	9.79 ± 0.08^a^
B	7.24 ± 1.00^d^	1.09 ± 0.23^a^	4.61 ± 0.54^b^	76.85 ± 1.00^a^	10.22 ± 0.08^a^
C	4.58 ± 1.00^a^	0.82 ± 0.23^a^	4.53 ± 0.54^b^	80.31 ± 0.08^b^	9.76 ± 0.08^a^
D	4.87 ± 1.00^b^	0.91 ± 0.23^a^	4.45 ± 0.54^b^	79.17 ± 0.08^b^	10.60 ± 0.08^a^

*Note*

The values represent the mean of the triplicate of each sample. Mean value with the same superscript across the same column is not significantly different at *p* ≤ 0.05.

This report was in accordance with Worthington (2001) which reported that nitrogen from every type of fertilizers influences the amount and quality of the plant‐produced protein. Maize treated with SB had the lowest protein content; this is in agreement with the value reported by Eltun (1996) who reported that organically grown cereals usually have significantly lower protein contents than the nonorganically grown cereals. This is also in agreement with previous reports (Mäder et al., 2007; Mazzoncini, Antichi, Silvestri, Ciantelli, & Sgherri, 2015) that reported that there was decrease in protein content in organically grown cereals compared with conventional cereals.

The ash content of the maize flour ranged between 0.82% and 1.09%. The percentage range of ash content of these maize flour samples was slightly lower than the range reported in the literature: Matt et al. (2011) reported ash content of maize in the range of 1.4%–3.3%. The variation may be attributed to environmental factors and agronomic practices. It was observed that maize planted with NPK fertilizer had the highest ash content, while maize planted with SB had the lowest ash content. There was no significant difference irrespective of the treatment methods applied between the maize samples. This result is in agreement with the result of Matt et al. (2011) which reported no differences in ash content between eight cultivars of organic and conventional durum wheat. The results showed that the use of biochar or NPK fertilizer as soil treatments did not affect the ash content of the maize. The fat composition of the maize samples ranged from 3.84% to 4.61%. It was observed that maize planted with NPK fertilizer had the highest crude fat 4.61%, while maize planted on normal soil had the least % crude fat. There was no significant difference in their crude fat content of maize treated on sawdust and GB soil.

Results showed that the use of NPK fertilizer or biochar treatment did not affect the crude fat content of the maize sample but the maize treated with NPK fertilizer had the highest crude fat content compared to maize planted on normal soil. The percentage crude fat obtained in this report was in agreement with other researchers (Matilda, Einar, Rune, & Kjarten, 1993; Ndukwe, Edeoga, & Omosun, 2005).

There was no significant difference (*p* < 0.05) among the samples in terms of the moisture content with maize treated with GB having the highest moisture content of 10.60%, while maize treated with SB had the lowest moisture content value of 9.76%. This result was similar to what was reported on moisture content of maize products (9%–19%) by Trabelsi, Kraszewski, and Nelson (1998). However, it was observed that the treatment of maize sample with normal soil, NPK fertilized soil, and biochar fertilized soil, respectively, resulted in no differences in moisture content. The low moisture content in maize sample treated with SB soil serves as an indication that it will have higher storability; this will minimize fungal contamination and spoilage of the maize flour.

There was significant difference (*p* < 0.05) in the carbohydrate content of the maize samples. There was variation in carbohydrate content among the maize samples. Maize treated with SB had the highest carbohydrate content value of 80.31% and maize treated with NPK fertilizer had the lowest carbohydrate content value of 76.85%. The percentage range of carbohydrate content is higher than the range reported in the literature: Wilson et al. (1999) reported a slightly higher carbohydrate content of about 72%–73%, while Mlay et al. (2005) reported a higher carbohydrate content of 73.3% of maize kernel. There was no significant difference among the maize sample treated with SB and maize planted on normal soil.

### 3.1 Minerals

The mineral content of the maize flour samples is presented in Table 4. The result showed that the sodium (55.65 mg/100 g), magnesium (35.87 mg/100 g), and iron (6.78 mg/100 g) of maize planted on normal soil were significantly higher than maize samples treated with NPK fertilized soil and biochar fertilized soil. This report is in agreement with what was reported by Abiose and Ikujenlola (2014) on the comparison of mineral content of quality protein maize and conventional maize. Similarly, maize sample treated with SB had the least value in the content of iron (1.16 mg/100 g) and magnesium (18.31 mg/100 g). Maize sample on normal soil had the highest calcium content value of 48.95 mg/100 g, while maize sample treated on GB had the lowest calcium content value of 0.10 mg/100 g. There was significance difference in the mineral content of maize sample treated with both NPK fertilized soil and biochar fertilized soil as compared to normal soil which may be as a result of concentration of exchangeable cation (such as Ca, Mg, and K) on the treated soil, thus affecting final mineral content of the maize samples.

**Table 4:** Mineral composition of maize flour

Samples	Na	Mg	K	Ca	Fe (mg/100 g)
A	55.65^c^ ± 1.00	35.87^d^ ± 1.00	10.07^a^ ± 0.28	48.95^c^ ± 1.00	6.78^d^ ± 1.00
B	0.01^a^ ± 0.97	24.86^b^ ± 1.00	10.30^a^ ± 0.28	8.85^b^ ± 1.00	1.86^b^ ± 1.00
C	0.16^a^ ± 0.97	18.31^a^ ± 1.00	11.83^b^ ± 1.00	2.10^a^ ± 0.16	1.16^a^ ± 1.00
D	25.00^b^ ± 1.00	29.57^c^ ± 1.00	13.05^c^ ± 1.00	0.10^a^ ± 0.16	3.13^c^ ± 1.00

*Notes*

GB: gliricidia biochar; SB: sawdust biochar.

Means in the same column with the same superscript are not significantly different at *p* < 0.05.

Key: A: Maize planted on normal soil (control); B: Maize planted on NPK fertilized soil; C: Maize planted on SB; D: Maize planted on GB.

## 4 CONCLUSION

The research work has been able to show the effect of soil treatments methods on the quality of maize samples. There is significant difference in the protein content of the treated maize samples, with maize soil‐treated with NPK fertilizer having the highest. There was no significant difference in ash, moisture, and crude fat contents in the maize samples. Mineral content of maize planted in control plot was higher than those on the amended soil. Maize samples fertilized with GB also had the highest quantity of essential minerals. However, SB and GB did not affect the proximate composition and mineral content of maize flour.

## CONFLICT OF INTEREST

All authors have no conflict of interest to report.

## AUTHOR CONTRIBUTION

A.M.: provided the conception and design of the study, acquisition of data, analysis and interpretation of data, and drafting the article, B.O.: revised it critically for important intellectual content and final approval of the version to be submitted; A.M. and J.A.: supplied the acquisition of data and drafting of manuscript; A.M., B.O., and J.A.: supplied the design of study, analysis and interpretation; M.A.: supplied the acquisition of data; B.O. and J.A.: were responsible for the article critically for important intellectual content; and B.O.: revised the article critically for important intellectual content and gave final approval of the version to be submitted.
